# Endogenous murine leukemia retroviral variation across wild European and inbred strains of house mouse

**DOI:** 10.1186/s12864-015-1766-z

**Published:** 2015-08-18

**Authors:** Stefanie Hartmann, Natascha Hasenkamp, Jens Mayer, Johan Michaux, Serge Morand, Camila J. Mazzoni, Alfred L. Roca, Alex D. Greenwood

**Affiliations:** Institute of Biochemistry and Biology, University of Potsdam, Karl-Liebknecht-Str 22-24, Potsdam, 14476 Germany; Department of Evolutionary Genetics, Max-Planck-Institute for Evolutionary Biology, August-Thienemann-Str. 2, Plön, 24306 Germany; Department of Human Genetics, Center of Human and Molecular Biology, Medical Faculty, University of Saarland, Building 60, Homburg, 66421 Germany; Centre de Biologie et de Gestion des Populations, Campus International de Baillarguet, CS 30016, Montferrier-le-Lez, 34988 France; Conservation Genetics Unit, Institute of Botany (B. 22), University Liège, Liège, 4000 Belgium; CIRAD TA C- 22 / E - Campus international de Baillarguet, Montpellier Cedex 5, 34398 France; Berlin Center for Genomics in Biodiversity Research, Königin-Luise-Str. 6-8, Berlin, 14195 Germany; Leibniz Institute for Zoo and Wildlife Research, Alfred-Kowalke-Straße 17, Berlin, 10315 Germany; Department of Animal Sciences, University of Illinois at Urbana-Champaign, 1207 W. Gregory, Urbana, 61801 IL USA

**Keywords:** Murine leukemia virus, Endogenous retrovirus, Xpr1, XMRV, Genomic evolution, Markov cluster algorithm

## Abstract

**Background:**

Endogenous murine leukemia retroviruses (MLVs) are high copy number proviral elements difficult to comprehensively characterize using standard low throughput sequencing approaches. However, high throughput approaches generate data that is challenging to process, interpret and present.

**Results:**

Next generation sequencing (NGS) data was generated for MLVs from two wild caught *Mus musculus domesticus* (from mainland France and Corsica) and for inbred laboratory mouse strains C3H, LP/J and SJL. Sequence reads were grouped using a novel sequence clustering approach as applied to retroviral sequences. A Markov cluster algorithm was employed, and the sequence reads were queried for matches to specific xenotropic (*Xmv*), polytropic (*Pmv*) and modified polytropic (*Mpmv*) viral reference sequences.

**Conclusions:**

Various MLV subtypes were more widespread than expected among the mice, which may be due to the higher coverage of NGS, or to the presence of similar sequence across many different proviral loci. The results did not correlate with variation in the major MLV receptor *Xpr1*, which can restrict exogenous MLVs, suggesting that endogenous MLV distribution may reflect gene flow more than past resistance to infection.

**Electronic supplementary material:**

The online version of this article (doi:10.1186/s12864-015-1766-z) contains supplementary material, which is available to authorized users.

## Background

Murine leukemia viruses (MLVs) are present in the germ line of the house mouse *Mus musculus* and of related species as endogenous retroviruses [1]. Many are inactive and transmitted vertically, but MLVs can also exist as horizontally transmitted exogenous retroviruses (ERVs). Because endogenous MLVs are highly variable in sequence and present in the genome at high copy number, a comprehensive analysis of their presence and distribution has generally been difficult: low throughput data sets generated by Sanger sequencing may only reveal a small proportion of the diversity. Many distinct MLVs are also similar enough so that PCR-based approaches may not be able to distinguish among them. Although using next generation sequencing (NGS) data can be effective for characterizing MLV diversity [2, 3], these datasets are often exceptionally complex, consisting of tens of thousands to many millions of sequence reads. These high-throughput data sets are not amenable to standard phylogenetic analysis, as there are substantial challenges for computing, evaluating, and visualizing alignments and phylogenies for such large data sets. In our analysis of NGS-generated data, we overcome these challenges by using a clustering approach to determine the distribution of MLVs in two wild-caught and three inbred laboratory strains of *M. musculus*. In addition, we also performed detailed sequence comparisons to determine the presence of specific viral reference sequences in these mice.

MLVs can be pathogenic, causing cellular transformation or leukemia, a cancer originating in the bone marrow and producing abnormal white blood cells. Different MLVs are able to infect different hosts, i.e., they have different host specificity: xenotropic MLV (*Xmv*) elements have a broad host range but are unable to infect laboratory mouse host strains, while polytropic MLV elements have a more restricted host range but are able to infect house mouse strains [4]. Polytropic MLVs can be phylogenetically subdivided into *Pmv* and modified polytropic retroviruses (*Mpmv*), which are genetically distinct but retain the same host specificity [4]. One recently reported xenotropic MLV, designated xenotropic like murine retrovirus (XMRV) was thought to be associated with prostate cancer and with chronic fatigue syndrome [5, 6]. It was subsequently demonstrated that detection of XMRV in cancer tissues was due to contamination of some molecular biology reagents with mouse genomic DNA [6] and that XMRV was actually a laboratory derived virus that originated from recombination in cell culture between two naturally occurring precursor viruses (PreXMRV-1 and PreXMRV-2, both endogenous retroviruses). XMRV then infected human tissues that had been co-cultured with mouse cells [5]. XMRV is the result of at least six recombination events between PreXMRV-1 and PreXMRV-2 [5] in mouse cells; this generated a virus that subsequently infected human cell cultures. The 3’ region of XMRV is generally homologous to the genome of a virus designated PreXMRV-1, while the 5’ region of XMRV is generally homologous to the 5’ region of PreXMRV-2 [5]. PreXMRV-1 and PreXMRV-2 are naturally occurring *Xmv*-like elements that are present in some but not all house mice [7].

Among exogenous MLVs, host range is affected by differences in the viral envelope protein that allow retroviruses to bind to host cellular receptors and enter host cells. Host range may also be affected by polymorphisms in the host receptor gene that codes for cellular receptors. In the case of MLVs, the host receptor is the xenotropic and polytropic retrovirus receptor 1 (XPR1) protein, an 8-transmembrane G protein-coupled receptor [8]. Non-synonymous variation in ECL 3 and 4 is associated with MLV *Pmv* and *Xmv* subtype restriction [9, 10]. Substitution of specific residues in ECL 3 is associated with xenotropic retroviral restriction *in vitro*. The *Xpr1* gene is polymorphic in mice, and specific alleles of *Xpr1* have been associated with restriction of the horizontal transfer of exogenous *Xmv*, *Pmv* or *Mpmv* retroviruses. For example the *Xpr1*^*n*^ allele allows infection of mouse cells by *Pmv* but not *Xmv* MLVs [11]. Exogenous retroviral restriction is thus strongly influenced by receptor differences in host cells. By contrast, endogenous MLVs are transmitted through vertical (parent-to-offspring) transmission, which could generate a phylogeographic pattern distinct from that of an infectious agent.

MLVs have previously been examined comprehensively primarily in the inbred laboratory mouse strain C57BL6/J yielding many groups of genetically distinct proviruses that are the result of infection of the germ lines of mice ancestral to C57BL6/J by various MLV lineages [1]. The presence and absence of retroviruses has generally been determined by Southern blot [12–14]. However, Southern blot may not be sensitive or specific enough to distinguish among closely related viruses or viruses that exist in low copy. Each individual in an inbred strain would be expected to carry the same fixed ERV integrations, although they could share different specific proviral loci depending on the laboratory strain genealogy [12–14]. By contrast, feral mice are from outbred populations where ERV insertional patterns will vary across individuals [15]. Absence of a specific proviral integration would not mean that a given mouse or mouse strain was free of a retroviral lineage, which could be present at other loci. In addition, *Xpr1* can only inhibit infection by exogenous retroviruses but cannot prevent the same viral lineages from being inherited as ERVs.

In order to comprehensively examine the presence or absence of *Xmv*, *Pmv* and *Mpmv*, we relied on Roche 454 FLX generated sequences of various MLV genome regions from different mice. We targeted five different regions of the MLV genome that cover the 6 putative recombination sites that generated XMRV from PreXMRV-1 and PreXMRV-2; these regions also allow *Xmv*, *Pmv* and *Mpmvs* elements to be distinguished from one another. These data allowed us to compare the distribution of proviral sequences identical or closely related to proviruses identified in C57BL6/J using low throughput methods, and to determine their distribution in wild mice. Our analyses show that various MLV subtypes are more widespread than expected among the mice, which may be due to the higher coverage of NGS, or to the presence of similar sequence across many proviral loci. The results were unrelated to variation in the major MLV receptor *Xpr1*, which can restrict exogenous MLVs, suggesting that endogenous MLV distribution reflects gene flow unrelated to exogenous infection.

## Results

### Mouse strains and MLV target regions

**Fig. 1:**
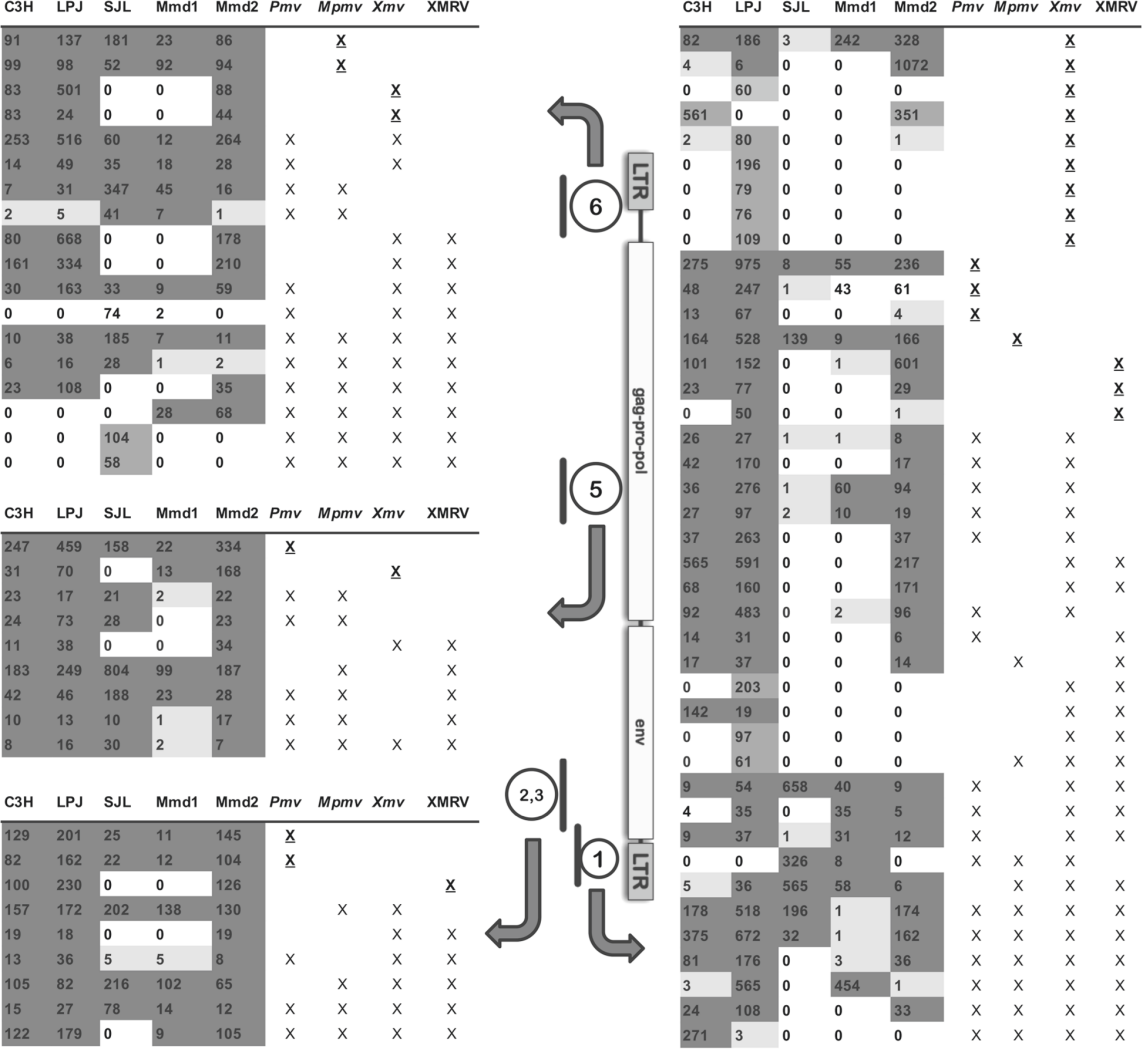
MLV regions sequenced and summary of sequence cluster information. The structure of the MLV genome is shown between two data tables, with the locations of retroviral regions that were amplified and sequenced indicated by the thick lines. The numbers with which these regions are labeled (1, 2,3, 5, 6) indicate the positions of the regions targeted by PCR, which covered 5 of the 6 recombination sites that created XMRV from PreXMRV-1 and PreXMRV-2 [5]. The target region labeled “2,3” was a single PCR product that included recombination sites 2 and 3. Note that there is no line segment numbered 4, since the PCR targeting the fourth recombination region yielded far fewer reads for all mice tested and was therefore excluded from further analyses. Block arrows point from the analyzed MLV regions to the corresponding table summarizing the clusters identified and analyzed for that genome region. Within the tables, each row represents one cluster of related sequences. A cluster is defined as sequences sharing sufficient identity with each other and with the chosen reference sequences to form a group distinct from other sequences. The first five columns in each table represent the number of sequences in a given cluster for the samples from inbred laboratory mouse strains C3H, LP/J, SJL and two wild caught mice Mmd1 (Corsica) and Mmd2 (mainland France). Shading of these cells correspond to the number of sequences per cluster that were identified per mouse: white for no sequences matching a cluster, light gray for 1-6 sequences, dark gray for more than 6 sequences. Cells shaded in intermediate gray indicates that a cluster was unique to a single mouse. The last four table columns list four different types of MLV (*Xmv*, XMRV, *Pmv* or *Mpmv*), each of which was compared to the mouse sequences generated by the current study. An “X” in these table cells indicates that one or more of the corresponding reference sequences were assigned to the given cluster. When only a single type of MLV reference sequence was assigned to the cluster, the “X” is underlined

MLV was examined in laboratory mouse strains C3H, LP/J and SJL, and in two wild caught *M. m. domesticus*; Mmd1 from the French island of Corsica and Mmd2 from mainland France. The inbred mouse strains C3H, LP/J and SJL were utilized because each strain exhibits multiple copies of *gag* leader sequences that resemble PreXMRV-2/XMRV, as had been previously determined using a DNA panel of laboratory and wild mice [3]. Thus, these strains were expected to carry xenotropic MLVs and *Xmv*-like elements. They also represent the major laboratory mouse groups: the C3H strain is part of the Lathrop/Castle lineage, the SJL strain belongs to the Swiss laboratory mouse lineage, and LP/J represents a third lineage of independent origin. The outbred mice represent two different feral populations, since gene flow is unlikely between mainland France and Corsica. The studied feral specimens correspond to the subspecies *Mus musculus domesticus* according to their distribution as well as based on previous phylogeographic studies performed on these animals [16]. Five regions of the MLV genome, each approximately 400 bp in length (total of approximately 1.6 kb), were amplified using PCR. One primer pair targeted part of the LTR (region 6, Fig. 1), while the other pairs each targeted one of the retroviral gene regions *gag, pol, env*, or the *env-* 3’LTR boundary (regions 5, 2, 3, and 1, respectively, Fig. 1). The amplicons also included previously identified recombination breakpoints for XMRV [5]. The relative positions of the amplified regions are shown in Fig. 1. PCR products were sequenced using GS FLX technology, which generated ca. 100,000 reads across the amplified MLV regions.

### Cluster analysis of MLV diversity

To determine the diversity of MLVs and their distribution in the different mice, we used the Markov Cluster Algorithm as implemented in the TRIBE-MCL software [17]. In this approach, sequences are grouped (“clustered”) based on pairwise similarity measures such as BLAST E-values [18]. Filtered NGS reads and selected reference sequences from the C57BL6/J genome of *Xmv, Pmv* and *Mpmv* ([4] were grouped into 7,041 sequence clusters, 5,815 of which were singletons. We further analyzed all clusters that contained at least 50 reads; smaller clusters were only considered if the clustering process assigned at least one of the reference sequences to it.

For each of these clusters, we determined which of the MLV target region it corresponded to. We also determined which mouse samples were represented in each of these groups. No cluster contained data from more than one MLV target region, which is as expected since each target region is in a different, non-overlapping part of the MLV genome (Fig. 1). The different regions of the MLV genome yielded quite different numbers of clusters, which was due to a combination of the number and variability of sequence reads per target region and per sample. Specifically, MLV target region 1 yielded the most clusters (n = 41; Fig. 1) and MLV target region 4 in the *env* gene the fewest (n = 4; data not shown). The number of clusters appeared to depend on the overall variability across MLVs at each genomic region targeted, with regions of greater variability generating a larger number of clusters (Additional file 1: Table S1). There were also different levels of sequence coverage per mouse, with the wild *M. m. domesticus* from Corsica (Mmd1) yielding the poorest coverage, and also displaying the lowest number of clusters. However, thousands of sequences were obtained for every mouse, and thus coverage for each target region was much higher than reported for Sanger sequence approaches [7]. Due to the relatively low coverage in general for MLV target region 4, located within the *env* gene, it was not included in subsequent analysis.

We then determined whether clusters shared identity with specific proviral insertion, such as have been classified for *Pmv, Mpmv* or *Xmv*. Sequences matching *Pmv* and *Mpmv* elements were generally found for each mouse for each MLV region targeted by PCR, and for XMRV in targeted regions 2, 3, and 1 (Fig. 1). Xmv sequence clusters were more variable regarding presence or absence, with many clusters absent in SJL and Mmd1 for all PCR products targeted (Fig. 1). The cluster profiles of SJL and Mmd1 were generally similar to each other but different from the C3H, LP/J and Mmd2 (Fig. 1). Most *Xmv* clusters were absent from SJL and Mmd1 for all PCR targeted regions. For *Xmv/XMRV* clusters, two were absent or rare for PCR target 6, one cluster for target 5, one cluster for target 2, 3 and six clusters for target 1. Although C3H, LP/J and Mmd2 were very similar in profiles, LP/J had five unique *Xmv* clusters one *Mpmv, Xmv, XMRV* and two *Xmv/XMRV* clusters in target region 6. Overall, the mice fell into two different groupings based on similarity of clusters: one grouping consisted of C3H, LP/J and Mmd2, which shared similar cluster profiles, and another group consisting of SJL and Mmd1.

### Assignment of sample sequences to *Xmv*, *Pmv* and *Mpmv* reference sequences

Although the clustering approach is an efficient way to get a broad overview of the similarities and differences of MLV sequences found in the mice, we also wanted to determine which of the specific MLVs (*Xmv, Pmv, Mpmv* elements) were present in which of the mice sampled. This analysis was independent of the assignment of sequences to clusters.

Each *Pmv, Mpmv* and *Xmv* provirus described in Jern et al. [1] is genetically distinct and can be distinguished from one or all of the approximately 400 bp PCR targeted regions in this study (Additional file 1: Table S1). Thus, when a sequence matched a specific proviral sequence we are not stating that the exact proviral insertion is present in a given mouse, but that the viral lineage that gave rise to that provirus is present.

For each of the *Xmv* (including the exogenous xenotropic MLV XMRV and its endogenous precursors PreXMRV-1 and PreXMRV-2), *Pmv* and *Mpmv* reference sequences reported previously [4, 7], we identified the sequence read in each sample that had the highest pairwise match to each of these reference sequences. This was done separately for each MLV target region. While it is clear that each endogenous retrovirus reported in Bamunusinghe et al. 2013 [4] represents a single fixed locus in C57BL6/J mice for a distinct retroviral element, such data does not indicate whether *Mus* in general was infected with identical or closely related strains with integration occurring elsewhere in the genome. Each of the retroviruses examined is genetically distinct (Additional file 1: Table S1). However, in some cases, even over 400 bp (the average sequence length targeted) some sequences are identical or are equally different from several proviruses. Such high identity proviruses are not distinct enough to examine individually by PCR based approaches that do not link all polymorphisms present in phase. The presence of a specific element was examined for each MLV target region based on sequence similarity to the characterized C57BL6/J ERV loci. A confounding factor for the *Pmv* and *Mpmv* groups was that, for several of the MLV regions targeted, the different reference proviruses shared very similar sequence identities (Additional file 1: Table S1). However, overall, individual elements could be distinguished by comparing all 4 regions for each retroviral lineage. It was also not possible to determine whether reads from different target regions represented the same or different proviral loci, as NGS approaches for sequencing PCR products over 1 kb with high accuracy were not yet commercially available at the time of sequence data generation.

To score a specific reference MLV as present in a mouse, we used a strict criterion of 100 % identity between a sequence read and the reference sequence. Generated MLV sequences had to match with 100 % identity to the reference virus for all of the MLV target regions, in order for the reference virus to be scored as present in a mouse. The *env* region with at least two-thirds lower coverage than for the other PCR products was removed from this analysis because the low coverage would likely bias the results to negative findings. However, upon scoring it, the results generally supported the results based on the remaining 4 PCR products. This scoring revealed the presence of *Pmv8, Pmv10* and *Pmv19*, which were identified in C3H and LP/J (Table 1). *Pmv14* was detected in C3H. *Pmv7, Pmv11* and *Pmv24* were detected in LP/J. Mmd2 carried sequences identical to *Pmv1, Pmv5, Pmv13, Pmv14, Pmv16, Pmv19* and *Pmv24*. SJL and Mmd1 did not carry any *Pmv* reference sequences under the criteria applied, except for *Pmv19* found in SJL. These results are consistent with the overall sequence clustering profiles (Fig. 1), in which SJL and Mmd1 tended to share one set of clusters, while C3H, LP/J and Mmd2 shared a different set of clusters and similarly lack or carry specific retroviral lineages.

**Table 1:** Maximum match between polytropic murine leukemia retrovirus (Pmv) and each next generation sequenced MLV region, in 5 mice

Reference	MLV region	C3H	LP/J	SJL	Mmd1	Mmd2
Pmv1	1	100	100	100	99.5	**100**
	2,3	100	100	99.7	99.7	**100**
	5	100	100	100	99.5	**100**
	6	99.8	99.8	99.8	99.8	**100**
Pmv5	1	100	100	100	99.5	**100**
	2,3	100	100	99.7	99.7	**100**
	5	100	100	100	99.5	**100**
	6	99.8	99.8	99.8	99.8	**100**
Pmv7	1	99.8	**100**	99.3	99.1	99.5
	2,3	100	**100**	100	100	100
	5	100	**100**	100	99.5	100
	6	100	**100**	100	99.8	100
Pmv8	1	**100**	**100**	96.4	99.3	99.8
	2,3	**100**	**100**	99.7	99.7	99.7
	5	**100**	**100**	100	99.5	100
	6	**100**	**100**	100	99.8	100
Pmv9	1	100	100	100	99.5	100
	2,3	99.7	99.7	99.5	99.5	99.7
	5	100	99.5	100	99.7	99.5
	6	99.3	99.3	99.5	98.8	99.3
Pmv10	1	**100**	**100**	99.5	99.1	100
	2,3	**100**	**100**	99.7	99.7	100
	5	**100**	**100**	100	99.5	100
	6	**100**	**100**	99.8	99.5	99.8
Pmv11	1	100	**100**	100	99.5	100
	2,3	100	**100**	99.7	99.7	100
	5	100	**100**	100	99.5	100
	6	99.8	**100**	99.5	99.5	99.8
Pmv12	1	100	100	99.8	99.3	100
	2,3	99.5	99.7	99.5	99.5	99.5
	5	100	100	100	99.5	100
	6	100	100	100	99.8	100
Pmv13	1	100	99.5	99.3	98.8	**100**
	2,3	100	100	100	100	**100**
	5	100	100	100	99.5	**100**
	6	99.8	99.8	99.5	99.5	**100**
Pmv14	1	**100**	99.8	99.5	99.3	**100**
	2,3	**100**	100	99.7	98.9	**100**
	5	**100**	100	99.7	99.2	**100**
	6	**100**	99.8	99.8	99.5	**100**
Pmv15	1	99.8	100	99.5	99.3	99.5
	2,3	99.5	99.5	99.5	99.5	99.5
	5	100	100	100	99.5	100
	6	100	100	100	99.8	100
Pmv16	1	100	100	100	99.5	**100**
	2,3	100	100	100	100	**100**
	5	100	100	100	99.5	**100**
	6	99.8	99.8	99.8	99.8	**100**
Pmv18	1	100	100	100	99.5	100
	2,3	99.5	99.7	99.5	99.5	99.5
	5	100	100	100	99.5	100
	6	99.8	100	99.8	99.5	99.8
Pmv19	1	**100**	**100**	**100**	99.5	**100**
	2,3	**100**	**100**	**100**	100	**100**
	5	**100**	**100**	**100**	99.5	**100**
	6	**100**	**100**	**100**	99.8	**100**
Pmv20	1	100	100	99.8	99.3	100
	2,3	100	99.5	99.5	99.2	99.5
	5	100	100	100	99.5	100
	6	99.8	100	99.5	99.5	100
Pmv21	1	99.5	99.8	99.3	98.8	99.5
	2,3	99.5	99.5	99.7	99.5	99.7
	5	100	100	100	99.5	100
	6	99.8	99.8	99.8	99.8	100
Pmv22	1	100	100	100	99.5	100
	2,3	99.7	99.7	99.7	99.7	99.7
	5	100	100	100	99.5	100
	6	99.8	100	99.8	99.5	99.8
Pmv23	1	99.3	100	99.5	98.6	99.1
	2,3	98.9	98.9	99.2	98.9	99.2
	5	99.7	100	99.7	99.2	99.7
	6	99.8	100	99.3	99.3	99.8
Pmv24	1	100	**100**	99.8	99.3	**100**
	2,3	100	**100**	100	100	**100**
	5	100	**100**	100	99.5	**100**
	6	99.8	**100**	99.5	99.5	**100**

The use of boldface indicates that sequences with 100% identity to a reference were detected for all MLV target regions in a mouse. The Pmv reference sequences are those of Bamunusinghe et al. [4]

C3H and LP/J both carried sequences identical to *Mpmv10* for all of the MLV genomic regions examined (Table 2). However, targeted region 5 could not be examined, as this region is deleted in the Mpmv10 reference sequence. C3H carried regions with 100 % identity to *Mpmv4*, while LP/J carried *Mpmv1* and *Mpmv7*, and Mmd2 carried *Mpmv9*. SJL and Mmd1 did not carry any *Mpmv* under the criteria used. It is possible that some mice carried elements that were similar to but not 100 % identical to a given *Mpmv*, and the clustering analysis suggests that such similar elements were present in all mice tested.

**Table 2:** Maximum match between polytropic murine leukemia retrovirus (Mpmv) and each next generation sequenced MLV region, in 5 mice

Reference	MLV region	C3H	LP/J	SJL	Mmd1	Mmd2
Mpmv1	1	99.8	**100**	99.5	99.3	99.5
	2,3	99.7	**100**	99.7	99.7	100
	5	100	*100*	100	99.7	100
	6	99.5	**100**	99.8	99.3	100
Mpmv2	1	99.8	99	98.8	98.6	100
	2,3	100	100	99.5	99.5	100
	5	100	100	100	99.7	100
	6	100	99.8	99.5	99.5	99.8
Mpmv4	1	**100**	99.8	99.8	99.5	100
	2,3	**100**	99.7	99.5	99.5	99.7
	5	*100*	99.7	99.7	99.5	100
	6	**100**	100	100	100	100
Mpmv5	1	99.5	99.3	99.3	99	99.3
	2,3	98.9	98.9	98.9	98.7	99.2
	5	99.7	99.7	99.7	99.5	99.7
	6	99.3	99.3	99.3	99.3	99.3
Mpmv6	1	100	100	99.3	98.8	99.3
	2,3	100	100	99.5	99.5	99.7
	5	100	100	99.7	99.7	99.7
	6	99.8	99.3	98.6	98.6	98.8
Mpmv7	1	99.5	**100**	99	98.8	99.8
	2,3	100	**100**	99.7	99.7	100
	5	100	*100*	100	99.7	100
	6	100	**100**	99.8	99.8	100
Mpmv8	1	99.3	99	99	98.8	99.3
	2,3	100	99.7	99.5	99.2	99.5
	5	100	99.7	99.7	99.5	100
	6	100	100	100	100	100
Mpmv9	1	99.8	99	98.8	98.6	**100**
	2,3	100	100	99.5	99.5	*100*
	5	100	100	100	99.7	*100*
	6	100	99.8	99.8	99.8	**100**
Mpmv10	1	**100**	**100**	99.5	99.3	99.5
	2,3	**100**	**100**	99.5	99.5	99.7
	5	**-**	**-**	-	-	-
	6	**100**	**100**	99.5	99	99.3
Mpmv11	1	100	100	100	99.8	100
	2,3	100	100	99.7	99.7	100
	5	99.7	99.7	99.7	99.5	99.7
	6	99.5	99.5	99.5	99.5	99.5
Mpmv12	1	100	99.3	99	98.8	99.8
	2,3	100	99.7	99.5	99.2	99.7
	5	100	100	100	99.7	100
	6	99.8	99.5	99.5	99.5	99.5
Mpmv13	1	99.8	99.8	99.8	99.5	99.8
	2,3	99.5	99.5	99.5	99.2	99.7
	5	99.5	99.5	99.5	99.2	100
	6	100	100	100	100	100

The use of boldface indicates that sequences with 100% identity to a reference were detected for all MLV target regions in a mouse. The use of italics indicates that more than one Mpmv sequence in a cluster was 100% identical. Mpmv reference sequences are those of Bamunusinghe et al. [4]. A dash indicates that for a target MLV region, the region is deleted in the reference sequence relative to other MLV sequences

*Xmv* elements have greater sequence variability than *Pmv* or *Mpmv* elements. This likely reflects a younger age and more frequent exogenous replication cycles of both endogenous and exogenous Xmvs that will tend to diversify elements at a much higher rate than stable endogenous elements that evolve at the relatively slower mutational rate of the mammalian host. Thus, the criteria for classifying a specific *Xmv* as present were made less stringent, so that sequences were judged to be a match if they were more similar to a specific Xmv reference than they were to any other reference sequence (Table 3 and Additional file 1: Table S1). For example, among the reads of MLV target region 1 in C3H, the closest match to the *Xmv17* reference sequence had 99.5 % identity. Among the reference *Xmv* sequences, the closest match had 96.5 % identity to *Xmv17*. Thus the sequence in C3H was scored as a slightly divergent *Xmv17* since the C3H sequence had a greater similarity to *Xmv17* than the percent similarity of any other reference sequence to *Xmv17*. In a few instances, a target region of the MLV genome was very similar across two or more reference *Xmvs*, e.g. *Xmv17* and *Xmv12* were highly similar in several of the MLV genomic regions sequenced, and thus both were scored as present (Additional file 1: Table S1), although it is possible that only one of the proviruses was actually present.

**Table 3:** Maximum match between xenotropic murine leukemia retrovirus (Xmv) and each next generation sequenced MLV region, in 5 mice

Reference	MLV region	C3H	LP/J	SJL	Mmd1	Mmd2
Xmv8	1	88.3	88.8	86.9	88.6	95.1
	2,3	98.7	99.7	97.1	99.5	99.5
	5	98.4	98.7	92.9	97.9	98.7
	6	97.1	99.5	90	90	95.5
Xmv9	1	96.1	94.2	87.1	88	98.4
	2,3	99.2	97.9	97.3	98.7	99.2
	5	98.9	99.2	93.4	98.4	99.2
	6	90.4	89.8	86	86.4	89.9
Xmv10	1	88.6	80.4	84.7	79.6	98.4
	2,3	98.4	99.5	96.8	99.2	99.2
	5	-	-	-	-	-
	6	-	-	-	-	-
Xmv12	1	99.5	98.5	77.5	88.2	98.5
	2,3	100	99.2	99.5	99.7	100
	5	98.9	99.5	94	98.9	100
	6	100	100	98.3	98.1	99.8
Xmv13	1	96.7	96.9	95.9	96.5	96.7
	2,3	98.1	99.2	96.5	98.9	98.9
	5	99.2	99.5	93.7	98.7	99.5
	6	97.6	99	90.3	90	95.2
Xmv15	1	90.8	91.1	72	81.3	91.3
	2,3	100	99.2	99.5	99.7	100
	5	98.9	99.5	94	98.9	100
	6	98.8	98.8	98.5	98.3	98.5
Xmv17	1	**99.5**	96.2	82.7	95.5	**99.5**
	2,3	**100**	99.2	99.2	99.2	**99.7**
	5	**-**	-	-	-	**-**
	6	**99.5**	99.5	97.9	97.6	**99.8**
Xmv18	1	99.5	98.2	77.5	88.2	**99.5**
	2,3	100	99.2	99.5	99.7	**100**
	5	98.9	99.5	94	98.9	**100**
	6	99.8	99.8	98.1	97.9	**100**
Xmv19	1	98.6	99.1	77.3	87.9	98.7
	2,3	100	99.2	99.5	99.7	100
	5	98.9	99.5	94	98.9	100
	6	52.2	46.9	48.5	48.9	49.8
Xmv41	1	96.8	97.5	85.4	95.1	97.3
	2,3	97.9	97.9	95.7	97.1	98.4
	5	96.3	96.6	93.4	93.1	96.8
	6	97.7	98.4	83.6	84.2	97.2
Xmv42	1	**99.1**	**99.1**	**98.6**	**98.8**	**99.5**
	2,3	**99.2**	**98.9**	**99.2**	**99.5**	**99.5**
	5	**-**	**-**	**-**	**-**	**-**
	6	**99**	**99.3**	**99**	**99**	**99.3**
Xmv43	1	98.3	98.5	86.3	96.3	**98.3**
	2,3	98.4	98.4	96.3	97.6	**98.9**
	5	98.7	99.2	93.7	98.7	**99.7**
	6	98.6	98.4	84.6	84.7	**98.4**

The use of boldface indicates that across all MLV target regions, the generated sequence read was more similar to the Xmv reference than were other Xmv references. Xmv reference sequences are those of Bamunusinghe et al. [4]. A dash indicates that for a target MLV region, the region is deleted in the reference sequence relative to other MLV sequences

Using the above criteria, *Xmv42* was identified in all individuals examined, and it was the only *Xmv* detected in SJL and Mmd1 (Table 3). *Xmv17* was found in C3H and Mmd2. Using similar criteria, there was evidence for the presence of the *Xmv* group PreXMRV-2 in all five mice tested (Table 4).

**Table 4:** Identity to XMRV-like sequences

	MLV Target	C3H	LP/J	SJL	Mmd1	Mmd2	PreXMRV1	PreXMRV2	XMRV
PreXMRV1	1	96.6	96.6	84.9	96.3	94.1	100	90.8	98.3
	2,3	99.7	99.2	92	99.2	95.2	100	91.2	92.3
	5	98.7	98.9	92.9	99.2	92.6	100	91.1	92.2
	6	96.5	97.4	83.4	96.5	83.3	100	78.7	86.5
PreXMRV2	1	**100**	**100**	**96.6**	**100**	**97.2**	90.8	100	92.4
	2,3	**100**	**100**	**99.5**	**100**	**99.5**	91.2	100	98.9
	5	**100**	**100**	**99.7**	**100**	**99.5**	91.1	100	89.8
	6	**99.5**	**100**	**95.4**	**99.7**	**95.1**	78.7	100	91.6
XMRV	1	97.3	97.3	86.5	97.3	95.6	98.3	92.4	100
	2,3	98.9	98.9	98.4	98.9	98.9	92.3	98.9	100
	5	95.8	95.8	92.8	95.5	92.6	92.2	89.8	100
	6	93.1	96.8	88.6	94.9	90	86.5	91.6	100

The use of boldface indicates that across all MLV target regions, a generated sequence read was more similar to the reference than were other XMRV references. Although in this table, PreXMRV1 could be predicted to be present in C3H, LP/J and Mmd1, Xmv43 exhibited higher identity to several breakpoints than the sequences obtained from the mice in this study. Thus, we cannot conclude that any of these mice carry PreXMRV1

The reference sequences *Xmv8, Xmv13, Xmv15, Pmv11, Pmv20, Mpmv2, Mpvm9* and *Mpmv12* had been derived from distinct proviral loci present in C57BL6/J mice, for which the integration sites are known. We examined if any of these specific previously characterized proviral sequences were present in our mouse DNA samples. This investigation was not meant to be comprehensive as the expectation, particularly for feral mice, was that identical proviral insertions would not be identified. Published primer pairs [4], with one primer based on the 5’ flanking region and one in the 5’ LTR, were used to determine if each individual proviral locus was present or absent in the mice. C3H, LP/J and SJL carried the integration site for *Pmv11*, in contrast with results reported in Frankel et al. 1989 [14]. C3H and SJL carried the *Pmv20* integration, consistent with Frankel et al. 1989 [14]. LP/J was positive for Xmv8 and SJL for *Pmv20*. None of the 5’ integration sites tested was identified as containing a provirus in either of the two wild mice, consistent with the absence of sequences with identity to these elements among the reads (data not shown). The exception was *Mpmv9*, which was present in Mmd2 (Table 2) suggesting that an identical provirus is located in a different genomic location in this feral mouse.

### Xpr1 haplotypes

The mouse *Xpr1* gene codes for the receptor for MLVs, which is an unusual G protein-coupled transmembrane protein with 8 transmembrane domains and four extracellular loops (ECLs) [8]. The C3H haplotype was similar to the *Xpr1*^*n*^ haplotype, which provides resistance to *Xmv* infection [19]. All other mice in this study carried an *Xpr1*^*svx*^ haplotype which is generally permissive to exogenous MLV infection. We note here that infection by an exogenous retrovirus involves binding to a host cell receptor. This is distinct from the spread of endogenous retroviruses which, in some cases, can be transmitted horizontally by infection if the proviral loci are capable of producing infectious virus but generally are transmitted vertically by inheritance. Sequencing of coding sequences for ECL 3, ECL 4 and Exon 4 in the 5 mice revealed that, relative to the other 3 mice, C3H and LP/J shared a haplotype in Exon 4 that changes an amino acid each at positions 103 (A/G) and 106 (A/T), with a synonymous substitution at position 105 (Table 5). C3H differed from LP/J and the other mice in ECL 3 by a unique non-synonymous substitution at position 500 (K/E). C3H had a unique ECL 4 sequence exhibiting a one amino acid deletion at position 583 and a unique substitution at position 590 (D/N). Thus, while LP/J and C3H were most similar to each other relative to the other mice in terms of cluster content, they still exhibited divergent *Xpr1* haplotypes. SJL, Mmd1 and Mmd2 shared the same Xpr1 haplotype, with the exception of a substitution at position 503 (K/N) in SJL relative to Mmd1 and Mmd2 (Table 5). Thus the *Xpr1* haplotype did not correspond to MLV cluster patterns, in which Mmd1 and SJL were similar in sequence cluster profile with a few exceptions across the MLV target regions, while Mmd2 exhibited a unique profile relative to SJL and Mmd1.

**Table 5:** Haplotype diversity of the Xpr1 gene across five mice

	Exon 4			ECL 3.2	ECL 3.3	ECL 4			
Residue no.	103	105	106	500	503	583	590		
amino acid	A/G	T	A/T	K/E	K/N	T/-	D/N		
SJL	C	A	G	A	A	A	G		
C3H	G	G	A	G	A	-	A		
LP/J	G	G	A	A	T	A	G		
Mmd1	C	A	G	A	T	A	G		
Mmd2	C	A	G	A	T	A	G		

ECL3 and 4 stand for the third and forth extracellular loop of *Xpr1*. The residue numbers indicate the positions in the primary sequence of the XPR1 protein, whereas the row below (amino acid) shows which kind of change occurs. The nucleotide changes are also shown below to give an impression on the amount of synonymous and nonsynonymous variation among the five analyzed mice. A dash stands for a missing nucleotide at the respective position relative to all other shown sequences

## Discussion

In this study we generated approximately 100,000 NGS reads covering five different proviral regions found in most MLVs. The approach applied here identified clusters of similar sequences that were present in just a single mouse from different mouse strains, as well as clusters and patterns of clusters that were shared across mice. For an inventory and description of retroviral variants based on NGS-derived sequence data, this approach had advantages over a conventional approach of aligning the generated sequence reads together with reference sequences, inferring a phylogeny, and analyzing the resulting clades with respect to the presence and absence of reference sequences and reads from specific samples of mice. Given sufficient computational resources [20], this type of standard phylogenetic analysis is possible using NGS-derived data sets consisting of thousands of sequence reads, although not without significant challenges. These include difficulties of aligning massive data sets to produce accurate phylogenies [21] and the interpretation of phylogenetic trees that are so large that individual clades are obscured and tracking individual samples is difficult. Clustering is computationally less taxing than alignment and tree building, and the results are easy to compare across mice (Fig. 1).

Results of clustering sequences showed that most of the MLV variation was in the LTRs, and thus the sequences from target regions 1 and 6 (which each included part of an LTR) formed the greatest number of clusters (Fig. 1). Overall, the C3H, LP/J and Mmd2 mice were similar among all the MLV target regions in the clusters they shared, while SJL and Mmd1 formed a second group (Fig. 1). These two groupings of mice are consistent with the patterns observed previously, when MLV insertional patterns were compared among mouse strains [4]. The dissimilar MLV sequences detected between Mmd1 (Corsica) and Mmd2 (mainland France) likely reflect the lack of gene flow between their populations. ERVs are transmitted by gene flow, their presence or absence depending on population structure. *Xpr1* allelic differences may strongly affect infectious exogenous MLVs, as replication would depend on successful cell entry by individual viruses. However, endogenous MLVs inherited genetically would not face cellular restriction by *Xpr1*.

XMRV was not identified in any sample, as expected of a virus that is a laboratory artifact. However, our results only partially overlap with work previously published on C3H examining specific integration sites by Southern blot [14]. *Pmv8*, *Pmv10*, and *Pmv14* were detected in C3H in both studies and *Pmv1, Pmv5, Pmv7, Pmv9, Pmv11, Pmv12, Pmv18, Pmv21, Pmv22* and *Pmv23* were absent in both studies, although in the case of *Pmv1* and *Pmv9*, distinguishing the individual ERVs was difficult from the results of Frankel et al. 1989 [14]. However, *Pmv13*, *Pmv15*, *Pmv20* and *Pmv24* were detected using Southern blot [14] but were not detected in our study under the criterion applied. *Pmv19* was detected in the present study but not found by Frankel et al. 1989 [14]. It should again be emphasized that Frankel et al. 1989 [14] determined the presence of specific ERV integrations, while the current study determines the presence or absence of a specific viral lineage. For C3H the results were in agreement with a previous study [12] for presence of *Mpmv10*. Similarly, *Mpmv1, Mpmv2, Mpmv5, Mpmv8*, and *Mpmv9* were absent in both data sets. In contrast, *Mpmv4* was present in the current study and *Mpmv6* and *Mpmv7* were identified in Frankel et al. 1990 [12] but not in the current study. The presence of *Xmv17* and the absence of *Xmv8, Xmv9, Xmv13, Xmv15*, and *Xmv41* is consistent between our study and previously reported results [13]. However, the absence of *Xmv12* and the presence of *Xmv42* in the current study are not.

Other findings were surprising in light of previous reports. SJL and Mmd1 both shared sequences resembling *Xmv42*, which was the only *Xmv* identified in these two mice. This is surprising as Southern blot hybridization and restriction fragment length results have previously suggested that *Xmv42* derived from *M. m. molossinus* [22], yet the current results showed it to be also present in feral *M. m. domesticus*. Substantial numbers of *Xmv*, *Pmv* or *Mpmv* elements were detected in the mice, whereas previous reports have suggested that these elements should be rare among European mice based on Southern blot and restriction digestion experiments [9], or based on analysis of specific loci known to carry *Xmv*, *Pmv* or *Mpmv* [4]. In fact, C3H, the only strain examined in common with the current study and previous work performed by Southern blot looking at specific ERV integrations while demonstrating some common sequences, contrasted in several cases for *Pmv*, *Mpmv* and *Xmv* elements. This extended to the PCR based amplification of two *Pmvs* where *Pmv20* was identified in both studies but *Pmv11* was only identified in the current study in C3H [14]. This suggests that C3H integrations may be polymorphic within the strain. These results also suggest determination of presence or absence of a specific ERV lineage cannot be achieved by examining specific integrations alone. Identical or closely related sequences may have entered individual mice or mouse lineages by separate integration events and thus, the same sequences may be located in different parts of the genome.

PreXMRV-2 was found in all samples. Using hybridization and integration-specific PCR, a previous study [7] suggested that none of these three types of mice should have co-occurring PreXMRV-1 and 2, and that European *Mus* would be expected to carry PreXMRV-2, consistent with the results here. In each case where the results may seem surprising, they may be attributed either to the much higher coverage provided by NGS, or to similar sequences being shared across many proviral loci. Even if a particular locus may not be present in a given mouse or population as established by Southern blot or locus-specific PCR, similar MLV sequences may be present across multiple loci. Thus strains and populations of mice are more likely to share similar sequences (common to many loci) than to share particular integration sites (single locus). As mentioned above, *Xpr1* alleles may effectively inhibit specific retroviral lineages from infecting cells when transmitted horizontally, but are ineffectual at inhibiting viral introgression when transmission is vertical.

## Conclusions

Cluster analysis of sequence data provided both computational and visualization advantages for a large and complex endogenous retroviral data set, compared to standard phylogenetic analysis. As much of the genome of multicellular species is composed of complex repetitive elements, this approach allowed us to analyze similar high-copy genomic elements even when identity among them is high. Analysis of sequence clusters and interrogation of the data with specific references revealed that MLV composition is highly variable among both inbred and wild mice. Elements identical or closely related to fixed integration sites in the C57BL6/J genome were found to be more widespread and variable in distribution in both laboratory mice and wild mice than expected. The discord between the MLV tropism determining *Xpr1* gene haplotypes and MLV distribution suggests that gene flow plays a more important role in MLV genomic colonization in mice than infection.

## Methods

### Mouse DNA

Genomic DNA from C3H/HeJ, LP/J, and SJL/J was kindly provided by John L. Goodier (McKusick-Nathans Institute of Genetic Medicine, Johns Hopkins School of Medicine, Baltimore MD, USA). The DNA had been originally obtained from the Jackson Laboratory. DNA from *Mus musculus domesticus* wild caught in Corsica (Mmd1) and mainland France (Mmd2) was generated as part of research by Johan Michaux and Serge Morand on mammals from the western Mediterranean islands [23, 24]. All animal experiments were performed according to the directive 2010/63/EEC on the Protection of Animals Used for Experimental and Other Scientific Purposes. The animal work also complied with French law (nu 2012Ű10 dated 05/01/2012 and 2013-118 dated 01/02/2013). The rodents, *Mus musculus domesticus*, were captured using Sherman traps and the study of mice did not require the approval of an ethics committee (European directives 86-609 CEE and 2010/63/EEC). *Mus musculus* is not protected, and no experiment was performed on living animals. No permit approval was needed as this species was trapped outside any preserve areas (national parks or natural reserves). The rodents were euthanized by vertebrate dislocation immediately after capture, in agreement with the legislation and the ethical recommendations (2010/63/EEC annexe IV) (see also protocol available on http://www.ceropath.org/references/rodent_protocols_book). All experimental protocols involving animals were carried out by qualified personnel (accreditation number of the Center of Biology and Management of the Populations (CBGP) for wild and inbred animal manipulations: A34-1691).

### PCR

Primer pairs for five MLV target regions were designed such that each primer pair generated PCR products of approximately 400 bp in length to match but not exceed the maximum read length of the GS FLX chemistry available at the time of sequence data generation. XMRV is the result of at least 6 recombination events between PreXMRV-1 and PreXMRV-2 [5] in mouse cells that infected human cell cultures. To avoid biasing the amplification for or against any one provirus type, all primers were designed in regions conserved in all known XMRV, PreXMRV-1 and PreXMRV-2 sequences and most MLVs in general. The primers were also designed so that the putative XMRV recombination crossover sites were in the middle of the PCR products, to maximize the number of informative differences up- and downstream of the crossovers. The four target regions on the MLV proviral genome for which sufficient coverage was obtained are shown in Fig. 1. Target position 2 included a region of the *gag* leader sequence containing a 24 bp deletion characteristic of XMRV and PreXMRV-2. Primer sequences were as follows: PCR product 1 (recombination site 1) (Forward 5’ ATT CTC AAC CGC TTG GTC CA 3’, Reverse 5’ TAA GGC TTG GGG TAT TTC CC 3’), PCR product 2 and 3 (recombination sites 2 and 3) (Forward 5’ AAA TCA GTC AGT GCC CTA GA 3’, Reverse 5’ TGA GTT GGT GAT ACT GTT GG 3’), PCR product 4 (crossover site 4) (Forward 5’ AGT TCC CAA AAC CCA TCA GG 3’, Reverse 5’ TTT TCT AAG GCC CCA AGG TC 3’), PCR product 5 (recombination site 5) (Forward 5’ AAG CAG GGC TAC GCC AAA GG 3’, Reverse 5’ TGG TCC GTG AGG TCC GGT CT 3’), PCR product 6 (recombination site 6) (Forward 5’ TCC TTG GGA GGG TCT CCT CA 3’, Reverse 5’ CGG TTT CGG CGW AAA ACC GA 3’). PCR was performed using Invitrogen Taq Polymerase using standard supplied buffers. Cycling conditions were 3 minutes 94 °C followed by 40 cycles of 30 sec 94 °C, 45 sec 54 °C and 45 sec 72 °C with a final 10 minute 72 °C extension. Water controls were always run as negative controls for PCR (data not shown). Contamination, especially from PCR reagents, was not detected at any point. Triplicate PCR products were pooled and purified using the QIAquick PCR Purification Kit (Qiagen).

### Sequencing

PCRs were performed in triplicate to minimize the inherent amplification bias of any given PCR of multicopy loci. The PCR products were verified by gel electrophoresis and, based on the intensity of the products, pooled in equal amounts for each of the three reactions. Each pool had a unique multiplex identifier (MID) (Roche Life Sciences) ligated to the products, which allowed for computational sorting of reads by animal post-sequencing. A 1/8 plate 454 FLX Titanium run was used to generate sequence data. The 454 sequence reads generated in this study were separated by MID using sfftools (Roche Life Sciences) for standard MIDs. Low quality reads were excluded from the analysis, resulting in a data set of approximately 103,761 reads.

### Xpr1 amplifications and sequencing

Five primer pairs were used to amplify and Sanger sequence several coding subregions of *Xpr1*. Primer sequences were as follows: exon 4 Forward 5’ GGG CCA AAA TGC TTT CTC TT 3’, Reverse 5’ TGA TTT CAA TCT TTA GAG GAT TCA GT 3’; ECL3.1 (part of exon 10) Forward 5’ TCC ATA AGG TAG GCT TTG CTG 3’, Reverse 5’ TCT TGG TTT ATG CTG GCA ATC 3’; ECL3.2 (exon 11) Forward 5’ CAC ACA CTG ATG GGG AGT TG 3’, Reverse 5’ GCA AAG TCC AGG AAA GCA GA 3’; ECL3.3 (part of exon 12) Forward 5’ TGG GCA CTA TGA AGA ATC CA 3’, Reverse 5’ GAG ACC CCA GTC CAT CTT GA 3’; ECL4 (part of exon 13) Forward 5’ AAC GCT TCT CCA TGA GTC TTT G 3’, Reverse 5’ GAT CAG ACT TGG TAT AAG TGT CT 3’. PCR was performed using the Qiagen Multiplex PCR Kit. For the reaction, 5 ng genomic DNA was applied to a reaction mix containing 1x Qiagen Multiplex PCR Mastermix and 0.2 *μ*M of each primer (Metabion) in a final volume of 10 *μ*l. The cycling conditions were 95 °C for 15 min followed by 40 cycles of 30 sec 95 °C, 1:30 min 60 °C, 1 min 72 °C with a final 10 min 72 °C extension. Water controls were run for each primer pair to control for contamination. An aliquot of the PCR product was visualized on a 1 % agarose gel, and the remaining product was purified. Cycle sequencing was carried out with the Big Dye Terminator v3.1 Cycle Sequencing Kit. For the sequencing, 1 *μ*l PCR product was used in a reaction mix of the standard kit supplies and 0.5 *μ*M primer in a final volume of 10 *μ*l. The cycling conditions were 96 °C for 1 min, followed by 25 cycles of 10 sec 95 °C, 15 sec 55 °C and 4 min 60 °C. Samples were purified by means of the BigDye XTerminator Purification Kit (Applied Biosystems) and then run on a 3730 DNA Analyzer (Applied Biosystems). Sequences were visualized and edited using CodonCode Aligner (CodonCode Corporation).

### Clustering analysis

For each mouse, cd-hit-est [25] was used to remove redundant reads at 100 % sequence identity, resulting in a reduction from 103,761 to 69,201 sequence reads. In addition, sequences shorter than 250 bp were removed, resulting in a final data set of 55,979 sequence reads. This data was combined with a set of 204 unique reference sequences from representative *Xmv*, *Pmv* and *Mpmv* MLVs (target region 1: 47 reference sequences, targets 2-3: 46, target 4: 37, target 5: 30, target 6: 44) into a single file and used to generate a matrix of pairwise BLASTN E-values [18]. The software Tribe-MCL [17] was then used to cluster sequences into families with an inflation value of 9. Tribe-MCL uses a Markov cluster (MCL) algorithm. In this approach, pairwise sequence similarity information for a set of sequences is used to construct a weighted graph, which is then converted into a Markov matrix. Next, simulation of stochastic flow in graphs is used to iteratively expand and inflate this matrix, with the goal of adjusting the edges until discrete and fully connected clusters are evident.

Sequence clusters that contained reference sequence matches for target regions 1, 2, 3, 4, 5 and 6 were directly used for further analysis. BLAST was used to assign reference sequences to all families with at least 50 sequences to which no reference was assigned during the clustering step. Specifically, each sequence in these families was compared to a database of the reference sequences, and the single best match with an E-value of at least 1-20 was recorded. This information was combined for each family, resulting in an assignment of reference sequences to families to which no reference was assigned during clustering.

### Assignment of sample sequences to specific reference sequences

For two separate sets of reference sequences (1. XMRV consensus, PreXMRV-1, PreXMR-2 [7]; 2. *Xmv*, *Pmv* and *Mpmv* sequences [4]), we computed the pairwise sequence identity among the reference sequences as well as between the reference sequences and the most similar sample sequence from each mouse. For the latter values, the single most similar sample sequence to each reference from each mouse for each MLV target region was first identified using BLASTN. Subsequently, pairwise identities were computed from pairwise optimal alignments using the *water* program of the EMBOSS package [26]. Computational analyses were implemented using custom Perl scripts that made use of BioPerl [27].

### Availability of supplementary material and data

Supplementary material is available as additional files through BioMed Central. The set of 55,979 sequence reads used for the analysis has been submitted to Dryad (http://datadryad.org).

## Additional file

Additional file 1
**Pairwise distances for all reference sequences.** For the PCR products 1, 2,3, 5, and 6, percentage of pairwise sequence identity was computed from optimal pairwise global alignments for the reference sequences from Kozak et al. [9] and the XMRV consensus, PreXMRV-1, and PreXMR-2 sequences. The XMRV consensus represents the majority consensus sequence of all avaialable XMRV sequences in GenBank for the regions covered by the PCR products. “DEL” indicates that for the specific provirus, this region of the genome is deleted in the region covered by the PCR product.

## Abbreviations

ERV: Endogenous Retrovirus
NGS: Next generation sequencing
*M. m. domesticus*: Mus musculus domesticus
Mmd1: *M. m. domesticus* from Corsica
Mmd2: *M. m. domesticus* from mainland France
MLVs: Murine leukemia retroviruses
Mpmv: Modified polytropic MLVs
Pmv: Polytropic MLVs
Xmv: Xenotropic MLVs
XMRV: Xenotropic like murine retrovirus
XPR1: Xenotropic and polytropic retrovirus receptor 1
