# Supplementary material for: Endogenous murine leukemia retroviral variation across wild European and inbred strains of house mouse
Source: BMC Genomics. 2015 Aug 18;16(1):613. doi: 10.1186/s12864-015-1766-z (PMC4538763; doi:10.1186/s12864-015-1766-z)

PCR product 1

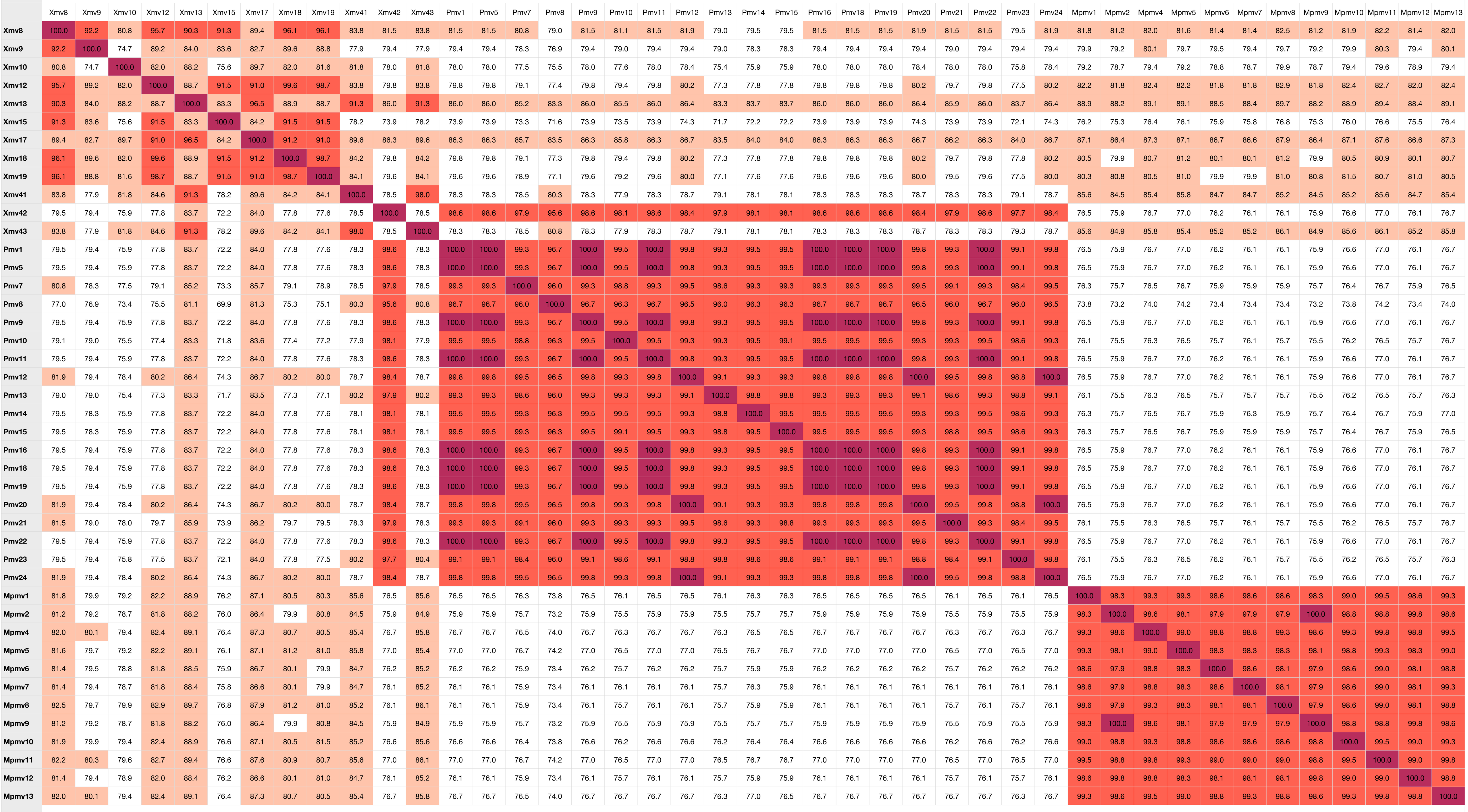

PCR product 2,3

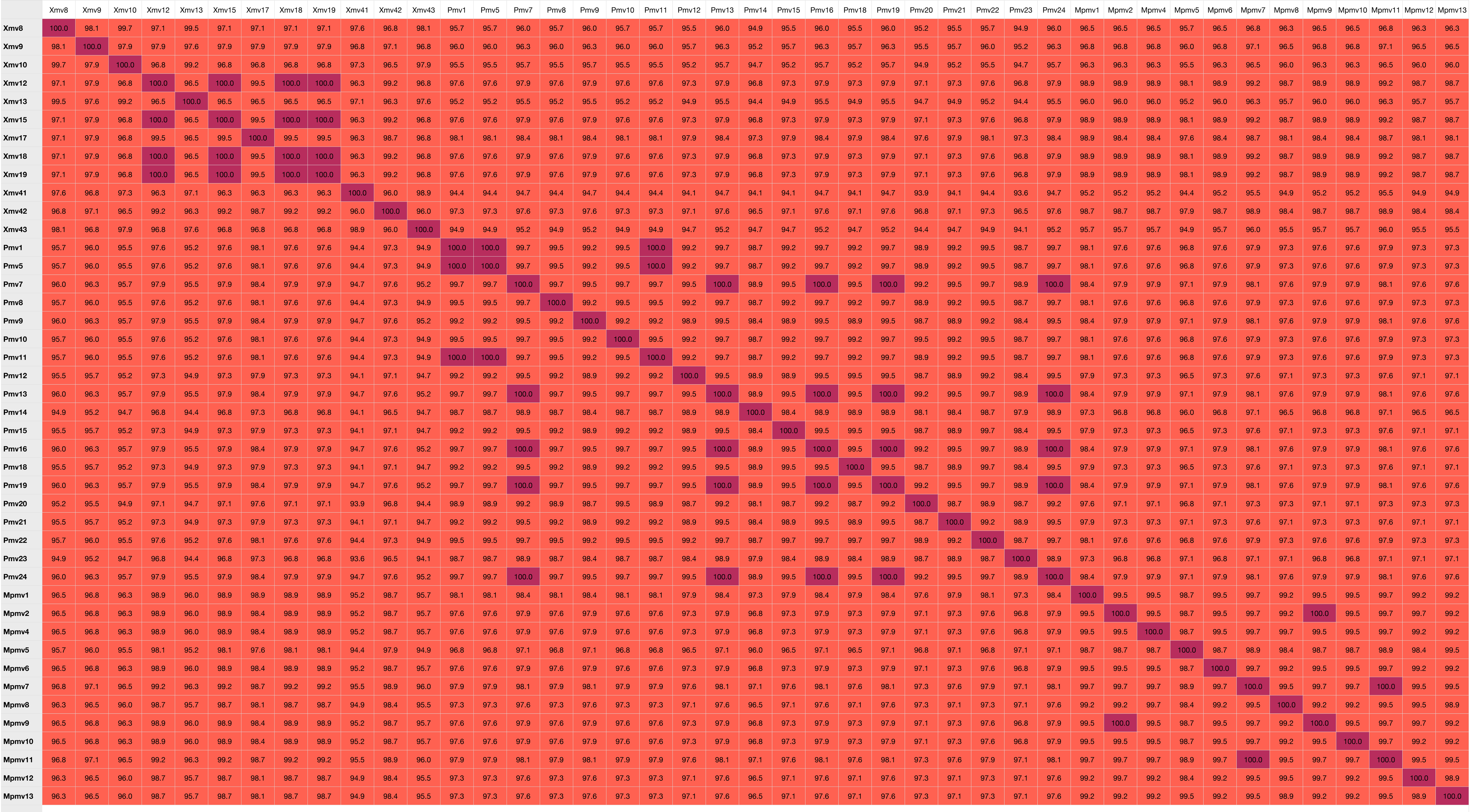

PCR product 5

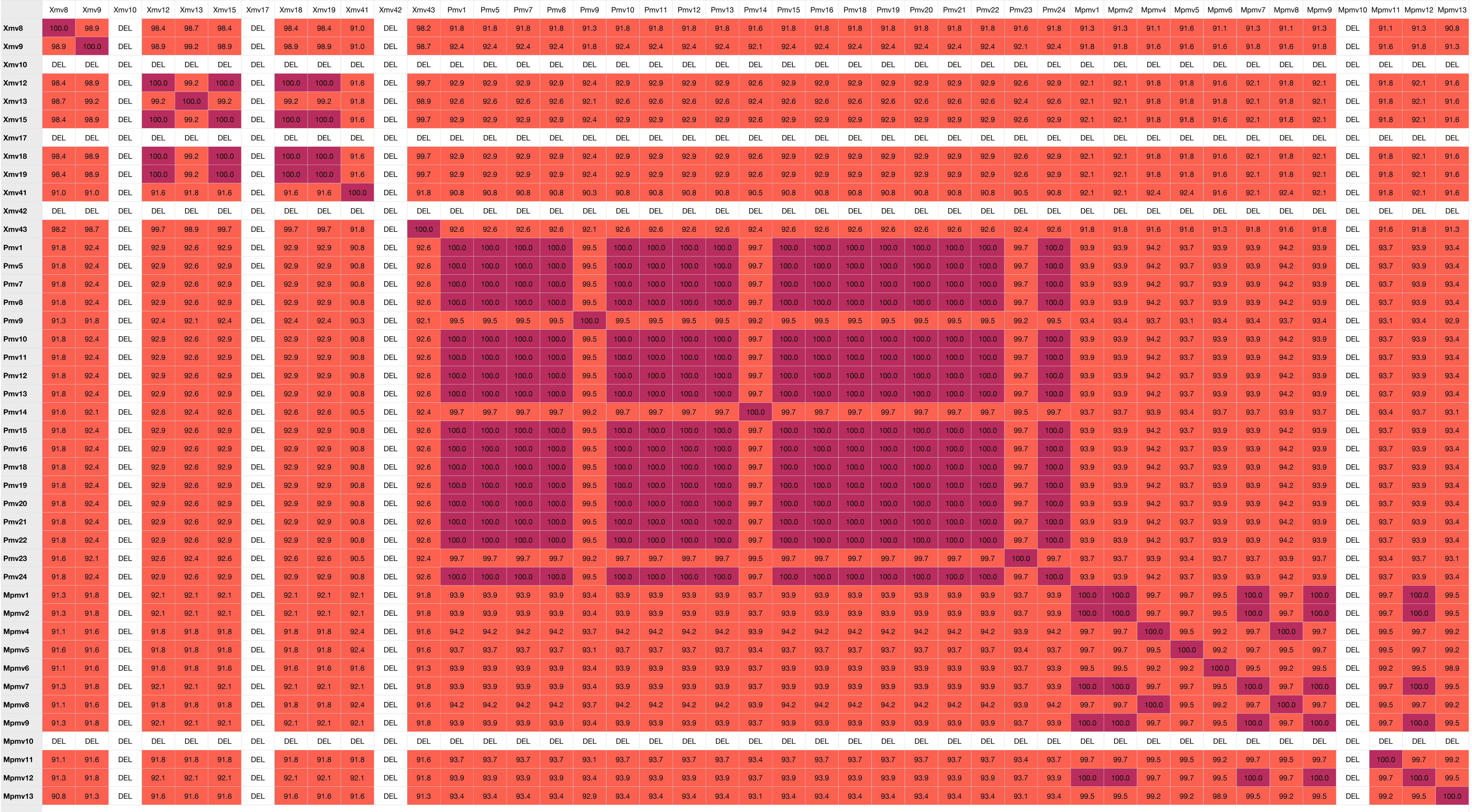

PRC product 6

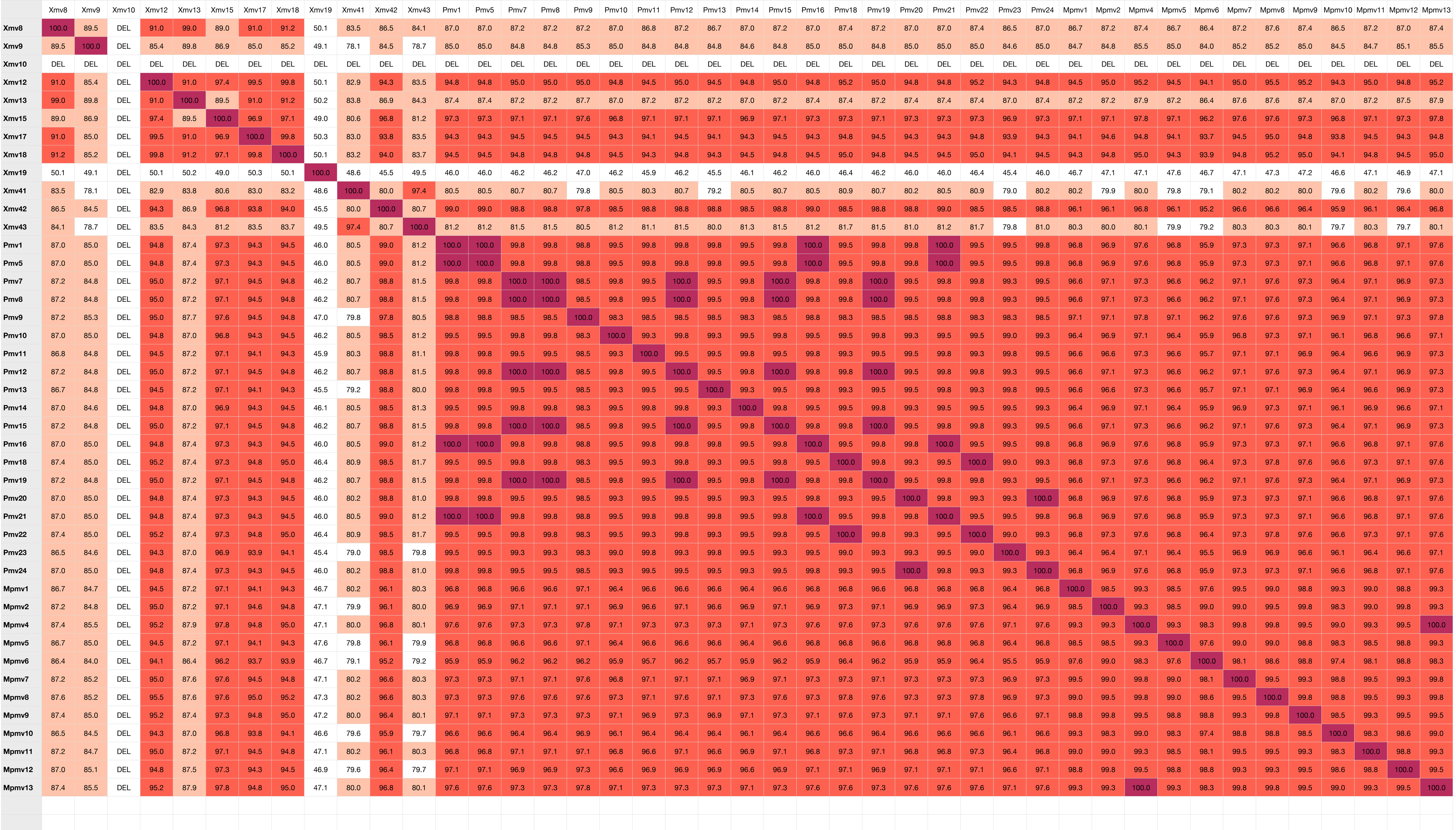

Supplement: Additional file 1 — Pairwise distances for all reference sequences. For the PCR products 1, 2,3, 5, and 6, percentage of pairwise sequence identity was computed from optimal pairwise global alignments for the reference sequences from Kozak et al. [9] and the XMRV consensus, PreXMRV-1, and PreXMR-2 sequences. The XMRV consensus represents the majority consensus sequence of all avaialable XMRV sequences in GenBank for the regions covered by the PCR products. “DEL” indicates that for the specific provirus, this region of the genome is deleted in the region covered by the PCR product. [file 12864_2015_1766_MOESM1_ESM.pdf]
